# Integrating Bulk and Single-Cell Transcriptomics with Machine Learning Reveals a Heme Metabolism-Based Panel for Lung Adenocarcinoma Chemotherapy Resistance

**DOI:** 10.3390/ijms26104685

**Published:** 2025-05-14

**Authors:** Lin Zhao, Haibo Han, Xuantong Zhou, Tongyang Gong, Yuge Zhu, Bufan Xiao, Shuchang Liu, Wei Zhao, Nan Wu

**Affiliations:** 1 Key Laboratory of Carcinogenesis and Translational Research (Ministry of Education), Department of Thoracic Surgery II, Peking University Cancer Hospital & Institute, Beijing 100142, China; 2 Key Laboratory of Carcinogenesis and Translational Research (Ministry of Education), Department of Clinical Laboratory, Peking University Cancer Hospital & Institute, Beijing 100142, China; 3 State Key Laboratory of Molecular Oncology, Beijing Key Laboratory of Carcinogenesis and Translational Research, Department of Thoracic Surgery II, Peking University Cancer Hospital & Institute, Beijing 100142, China

**Keywords:** heme metabolism, ferroptosis, chemotherapy resistance, lung adenocarcinoma, precision medicine, prognosis

## Abstract

Lung adenocarcinoma (LUAD) is a leading cause of cancer-related mortality, with heme metabolism playing a critical role in tumor progression and treatment resistance. This study investigates the clinical implications of heme metabolism in LUAD, focusing on its link to ferroptosis and drug sensitivity. Using multi-omics data from TCGA-LUAD, GEO databases, and a single-cell RNA-seq cohort, we identified two molecular subtypes based on heme metabolism-related genes. We further developed a prognostic panel, termed the heme metabolism risk score (HMRS), using LASSO and multivariate Cox regression analyses. The HMRS panel effectively stratified patients into high- and low-risk groups, with high-risk patients showing enhanced tumor proliferation, suppressed ferroptosis, and resistance to chemotherapy. Single-cell analysis revealed elevated heme metabolism risk in epithelial cells correlated with tumor progression. Drug sensitivity predictions were validated in platinum-based chemotherapy cohorts, confirming HMRS as a robust prognostic tool. ABCC2 was identified as a key regulator of ferroptosis and cisplatin resistance, with in vitro experiments demonstrating that ABCC2 knockdown enhanced cisplatin-induced ferroptosis. These findings highlight HMRS as a critical tool for patient stratification and ABCC2 as a promising therapeutic target to overcome cisplatin resistance.

## 1. Introduction

Lung adenocarcinoma (LUAD) represents one of the most prevalent and lethal malignancies worldwide, posing a significant public health challenge [1,2]. Emerging evidence highlights the critical role of tumor cell metabolism in disease progression and treatment response [3]. Among these metabolic pathways, heme metabolism has been shown to significantly influence tumor initiation, proliferation, metastasis, and energy metabolism, as well as modulate therapeutic sensitivity [4,5,6,7]. While heme metabolism has begun to attract interest in non-small-cell lung cancer (NSCLC), most studies have focused on individual molecules rather than the coordinated regulation of the entire pathway. Additionally, although epidemiological data suggest a link between dietary heme intake and lung cancer risk [8,9], large-scale population studies for patient stratification are still lacking. These gaps have hindered the clinical translation of heme metabolism research in prognosis and treatment decision-making.

Ferroptosis, an iron-dependent cell death process driven by lipid peroxidation, enables tumor cells to develop chemoresistance and survive under stress conditions [10,11,12,13,14]. Emerging evidence highlights a crucial mechanistic link between heme metabolism and ferroptosis regulation. This occurs through two primary mechanisms: (1) heme synthesis and degradation regulate the labile iron pool, with heme oxygenase-1 (HO-1)-mediated degradation releasing free iron that potentiates ferroptosis [15], while synthesis incorporates iron into protoporphyrin IX [16]; and (2) heme maintains mitochondrial electron transport chain integrity by promoting the expression [17,18] and proper assembly of protein complexes [19]. Insufficient heme levels impair these processes, resulting in electron leakage, elevated ROS, and subsequent lipid peroxidation. These mechanistic insights underscore the therapeutic potential of targeting heme metabolism in NSCLC. Investigating heme metabolism across NSCLC subtypes not only uncovers population-specific molecular differences but also provides novel therapeutic targets, paving the way for personalized treatment strategies tailored to distinct patient subgroups.

**Figure 1:**
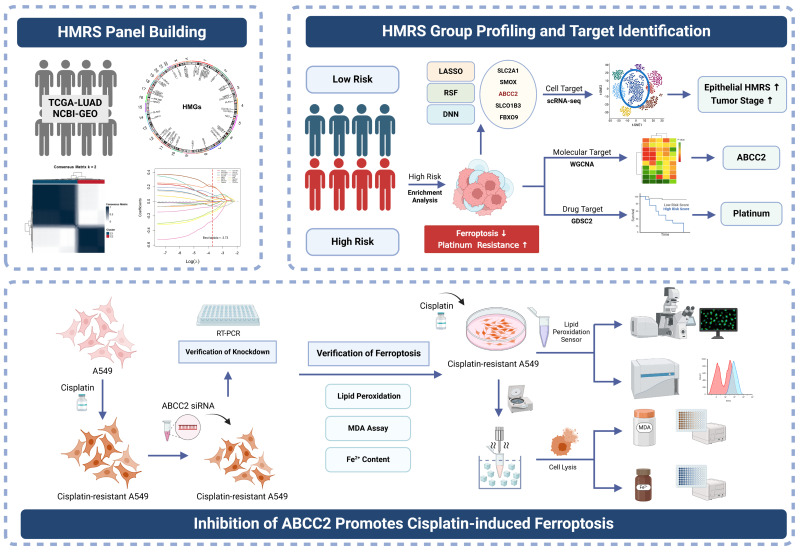
Workflow. Created in BioRender. Zhao, L. (2025) https://app.biorender.com/illustrations/6788bf4fc833ad2a7efad636 (accessed on 6 May 2025).

In this study, we systematically investigated heme metabolism-related genes (HMGs) in LUAD, establishing a heme metabolism risk score (HMRS) as a novel panel for prognosis and chemotherapy prediction. Using HMGs identified from the molecular signatures database (MsigDB), we identified prognostic markers through Cox regression and then developed HMRS using LASSO regression to stratify patients. Random survival forest (RSF) and deep neural network (DNN) analyses revealed five core HMGs, with high-risk groups showing enrichment in ferroptosis suppression and platinum-resistance pathways. Multi-omics analyses demonstrated the following: (1) progressive HMRS elevation in epithelial cells during disease progression (scRNA-seq); (2) ABCC2’s involvement in ferroptosis regulation (WGCNA); and (3) increased platinum resistance in high-risk patients (GDSC2). Functional validation in cisplatin-resistant A549 cells confirmed that ABCC2 knockdown enhances cisplatin-induced ferroptosis. Collectively, our findings establish HMRS as a clinically relevant biomarker and reveal ABCC2’s novel role in modulating cisplatin sensitivity through ferroptosis regulation. The workflow is shown in Figure 1.

## 2. Results

### 2.1. Heme Metabolism-Based Clusters Predict Prognosis in LUAD

**Figure 2:**
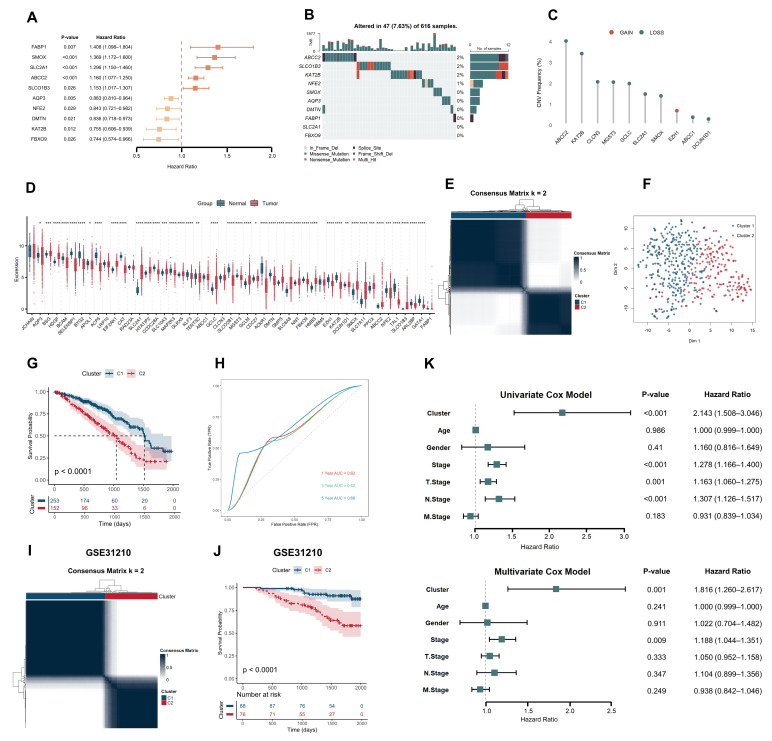
Heme metabolism-based clusters predict prognosis in LUAD. (**A**) Forest plot of HMGs: 49 prognostic genes were identified as significantly associated with overall survival by univariate Cox regression analysis; only 10 genes are shown in the figure. The vertical dashed line at HR = 1.0 indicates the reference line for no effect; values to the right suggest increased risk, while values to the left indicate protective effects. The position of each colored square represents the mean hazard ratio (HR) for the corresponding gene, while the horizontal lines denote the 95% confidence intervals; (**B**) Distribution of HMGs and mutation frequency in the TCGA-LUAD cohort; (**C**) CNV alteration frequency of HMGs in LUAD; (**D**) Differential expression of 49 HMG in LUAD tumors and normal tissues. * *p* < 0.05, ** *p* < 0.01, *** *p* < 0.001, **** *p* < 0.0001; (**E**) Consensus heatmap matrix of the TCGA-LUAD cohort; (**F**) T-SNE analysis showed significant differences between the two subtypes; (**G**) Kaplan–Meier survival analysis distinguished the prognosis of TCGA-LUAD patients based on cluster classification. The dashed lines indicate the median survival time, defined as the point where the survival probability is 50%. All dashed lines in the Kaplan–Meier plots throughout this study represent the same definition; (**H**) Time-dependent ROC curve analysis of TCGA-LUAD cohort. The gray dashed diagonal line represents the reference line for random classification (AUC = 0.5). All dashed diagonal lines in the time-dependent ROC curves throughout this study carry the same meaning; (**I**) Consensus heatmap matrix of the GSE31210 cohort; (**J**) Kaplan–Meier survival analysis distinguished the prognosis of GSE31210 patients based on cluster classification; and (**K**) Unifactorial and multifactorial demonstration of clinicopathologic factors and heme metabolism-based clusters.

Through a comprehensive screening of the molecular signatures database (MSigDB), we identified 282 heme metabolism-related genes (HMGs) (Table A1). In the TCGA-LUAD cohort, univariate Cox regression analysis of these 282 genes revealed 49 prognostic genes strongly associated with overall survival (OS) (Table A2). We present a forest plot of 10 of these genes. (Figure 2A). Further analysis of these genes demonstrated distinct mutation rates, with ABCC2 showing the highest mutation frequency, followed by SLCO1B3 and KAT2B (Figure 2B). Additionally, significant differences in DNA copy number variation (CNV) were observed among these genes, particularly with ABCC2 and KAT2B exhibiting higher CNV depletion (Figure 2C). The differential expression analysis of HMGs between normal and tumor tissues further revealed that the majority of these genes were significantly dysregulated in LUAD tumor tissues, highlighting their potential roles in tumor progression (Figure 2D).

**Figure A1:**
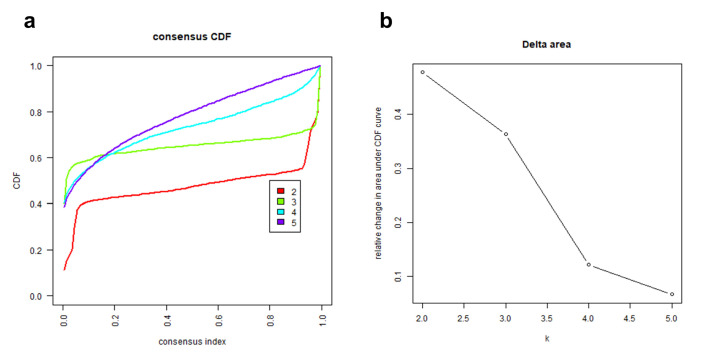
Selection of optimal k value for consensus clustering. (**a**) The CDF curve for k = 2 exhibits the flattest profile, indicating the most stable and consistent clustering results; (**b**) Selection of optimal k value based on delta area analysis where the delta area curve shows a significant change at k = 2, indicating that the clustering stability is optimal at this value.

**Figure A2:**
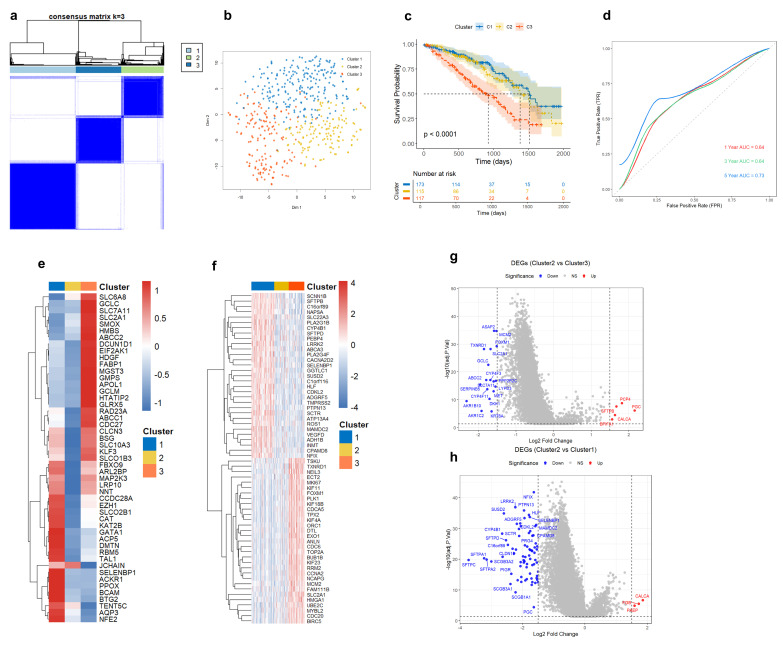
(**a**) Consensus heatmap matrix of the TCGA-LUAD cohort (k = 3); (**b**) T-SNE analysis showed significant differences between the three subtypes; (**c**) Kaplan–Meier survival analysis distinguished the prognosis of TCGA-LUAD patients based on cluster classification; (**d**) Time-dependent ROC curve analysis of TCGA-LUAD cohort; (**e**) Expression characteristics of 49 HMGs across different clusters; (**f**) Heatmap showing the expression patterns of a combined set of 90 top differentially expressed genes (DEGs), selected from pairwise comparisons (30 DEGs each) between clusters C1 vs. C2, C1 vs. C3, and C2 vs. C3; (**g**) Volcano plots of differential gene expression analyses for each pairwise comparison among C2 vs. C3. The horizontal dashed line represents the threshold for statistical significance (adjusted *p*-value = 0.05), and the vertical dashed lines indicate log_2_ fold change thresholds (±1.5), which define biologically meaningful changes in gene expression; (**h**) volcano plots of differential gene expression analyses for each pairwise comparison among C2 vs. C1.

Using the 49 HMGs, we performed unsupervised consensus clustering of TCGA-LUAD tumor patients to identify heme metabolism-associated subtypes. Based on the CDF and delta area plots, both k = 2 and k = 3 were considered appropriate (Figure A1a,b). While k = 3 demonstrated acceptable clustering stability and showed potential for subtype stratification (Figure A2), k = 2 provided clearer separation and was ultimately selected as the optimal choice, dividing the cohort into two clusters: C1 (*n* = 253) and C2 (*n* = 152) (Figure 2E). The rationale for k = 3 selection is discussed in detail in the Discussion Section. T-distributed stochastic neighbor embedding (t-SNE) confirmed clear separation between the two clusters (Figure 2F). Kaplan–Meier survival analysis revealed that patients in the C2 cluster exhibited significantly worse overall survival compared to those in the C1 cluster (*p* < 0.0001, Figure 2G). This finding was further validated in the GSE31210 dataset (Figure 2I,J). Time-dependent receiver operating characteristic (ROC) curve analysis further supported the predictive capability of the consensus clustering, with areas under the curve (AUCs) of 0.62, 0.62, and 0.68 at 1, 3, and 5 years, respectively, in the TCGA-LUAD training set (Figure 2H). Finally, univariate and multivariate Cox regression analyses confirmed that cluster grouping served as an independent prognostic factor for LUAD (Figure 2K).

### 2.2. HMRS Panel Demonstrates Robust Prognostic Utility in LUAD Risk Stratification

**Figure 3:**
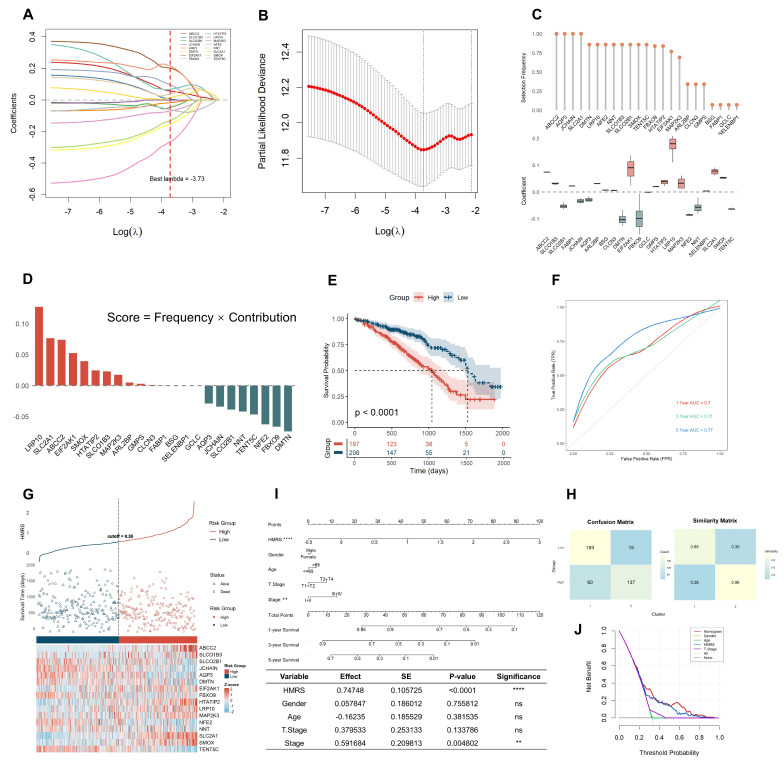
HMRS panel demonstrates robust prognostic utility in LUAD risk stratification. (**A**) Ten-fold cross-validation of parameter selection adjusted by LASSO regression from the 100th iteration, only 16 out of the 49 HMGs that were selected for further analysis are displayed in the coefficient paths plot for clarity and visual appeal; (**B**) Screening of coefficients under LASSO analysis, the optimal lambda value determined as 3.73 from the 100th iteration; (**C**) Frequency and regression coefficients of genes with a selection frequency greater than zero; (**D**) Selection of genes based on scoring metric where 16 genes with scores ranking in the top 50% were selected for further analysis; (**E**) Kaplan–Meier survival analysis distinguished the prognosis of TCGA-LUAD patients based on the HMRS panel; (**F**) Time-dependent ROC curve analysis of TCGA-LUAD cohort based on the HMRS panel; (**G**) Distribution of HMRS, survival time, and expression patterns between low- and high-risk groups; (**H**) Assessment of the consistency between cluster-based classification and HMRS risk group stratification through confusion matrix and Jaccard similarity index; (**I**) Nomogram combining age, grade, sex, T-stage, total stage, and HMRS. ns = not significant, ** *p* < 0.01, **** *p* < 0.0001; (**J**) DCA decision curve.

To develop a prognostic signature panel associated with heme metabolism, we utilized the aforementioned 49 HMGs and refined a panel through LASSO regression combined with multivariate Cox proportional hazards analysis (Figure 3A). Through 100 iterations of 10-fold cross-validation, the optimal lambda value was identified as 3.73. (Figure 3B). We found that four genes (ABCC2, AQP3, JCHAIN, and SLC2A1) were consistently selected (selection frequency = 1.00). All genes with a selection frequency greater than zero and their corresponding regression coefficients are displayed in Figure 3C, where higher absolute values indicate a greater contribution of the gene to the model. Additionally, using a scoring metric (score = frequency × contribution), we identified 16 genes (ABCC2, SLCO1B3, SLCO2B1, JCHAIN, AQP3, DMTN, EIF2AK1, FBXO9, HTATIP2, LRP10, MAP2K3, NFE2, NNT, SLC2A1, SMOX, and TENT5C) with scores ranking in the top 50% for further analysis (Figure 3D). Among these, LRP10, SLC2A1, and ABCC2 showed the strongest associations with patient survival events. Based on these findings, we established the heme metabolism risk score (HMRS) for each LUAD patient as a panel using the following formula: HMRS = ABCC2 × 0.0738 + SLCO1B3 × 0.0307 + SLCO2B1 × (−0.0524) + JCHAIN × (−0.0341) + AQP3 × (−0.0289) + DMTN × (−0.0999) + EIF2AK1 × 0.0880 + FBXO9 × (−0.0941) + HTATIP2 × 0.0345 + LRP10 × 0.1718 + MAP2K3 × 0.0365 + NFE2 × (−0.0848) + NNT × (−0.0567) + SLC2A1 × 0.0764 + SMOX × 0.0530 + TENT5C × (−0.0631).

**Figure A3:**
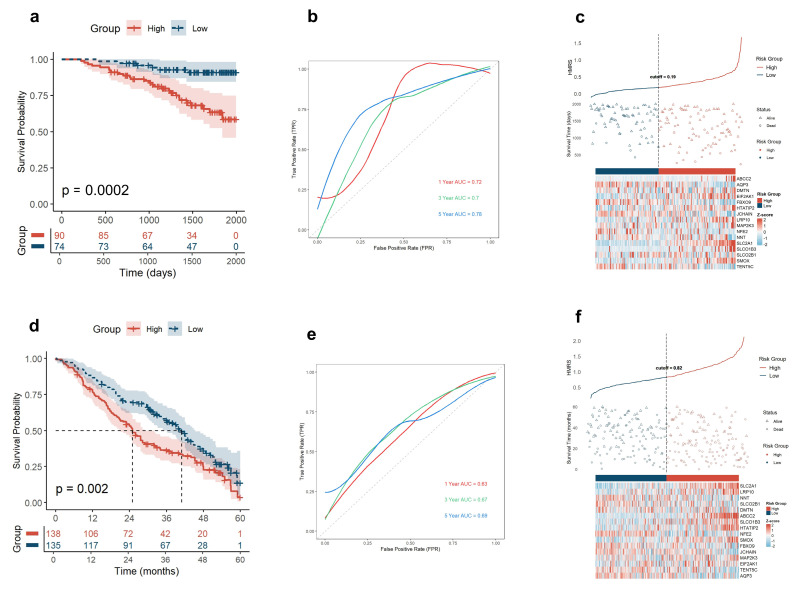
Validation cohort analysis of the HMRS panel. (**a**–**c**) Kaplan–Meier survival curves, time-dependent ROC curves, HMRS distribution, survival time, and expression patterns between low- and high-risk groups in the GSE31210 validation cohort; (**d**–**f**) Kaplan–Meier survival curves, time-dependent ROC curves, HMRS distribution, survival time, and expression patterns between low- and high-risk groups in the GSE68465 validation cohort.

Patients were then stratified into high-risk and low-risk groups based on the median HMRS value as the cutoff threshold. Kaplan–Meier survival analysis revealed that patients in the high-risk group had significantly shorter OS time compared to those in the low-risk group (*p* < 0.0001, Figure 3E). In the TCGA-LUAD training cohort, the panel achieved AUC values of 0.70, 0.71, and 0.77 for predicting 1-, 3-, and 5-year survival rates, respectively (Figure 3F). Consistent predictive performance was observed in two independent validation cohorts, with AUC values of 0.72, 0.70, and 0.78 in the GSE31210 cohort and 0.63, 0.66, and 0.69 in the GSE68465 cohort (Figure A3a–f). Notably, the panel not only demonstrated robust predictive stability in time-dependent ROC analysis but also outperformed consensus clustering methods in risk stratification. These findings highlight the effectiveness of the HMRS panel in distinguishing prognostic risk among LUAD patients, offering a reliable quantitative tool for precise prognostic evaluation. The heatmap revealed the differential expression patterns of the 16 heme metabolism risk genes between the high-risk and low-risk groups (Figure 3G). Prominently, the z-score values of ABCC2, SLCO1B3, SLC2A1, and SMOX were significantly higher in the high-risk group compared to the low-risk group, suggesting their potential pivotal roles in determining poor prognosis.

To assess the consistency between consensus clustering and LASSO-based risk stratification, we employed the Jaccard similarity measure. The analysis revealed a high degree of similarity between the two classification methods, with a Jaccard index of 0.96 between the high-risk group and Cluster 2 and 0.85 between the low-risk group and Cluster 1 (Figure 3H). These results indicate strong concordance between consensus clustering and LASSO-based clustering, particularly in the high-risk prognosis group, which exhibited nearly identical patient distributions. This high consistency suggests that patients in the high-risk group and Cluster 2 may share similar biological characteristics, potentially leading to comparable disease progression patterns, treatment responses, and clinical outcomes.

To enhance the clinical utility of the heme metabolism risk score (HMRS) panel, we constructed a nomogram integrating HMRS panel with other clinical characteristics, providing a comprehensive and intuitive risk assessment tool for LUAD patients (Figure 3I). Comparative analysis demonstrated that HMRS panel exhibited superior predictive performance over other clinical factors. Decision curve analysis (DCA) further confirmed that the nomogram, combining the HMRS panel with clinical features, significantly outperformed models based solely on clinical characteristics in terms of clinical predictive utility (Figure 3J). These findings suggest that the HMRS panel may have robust prognostic predictive potential in assessing LUAD patient risk.

### 2.3. Metabolic Reprogramming and Ferroptosis Regulation Are Key Differences in HMRS-Based Groups

**Figure 4:**
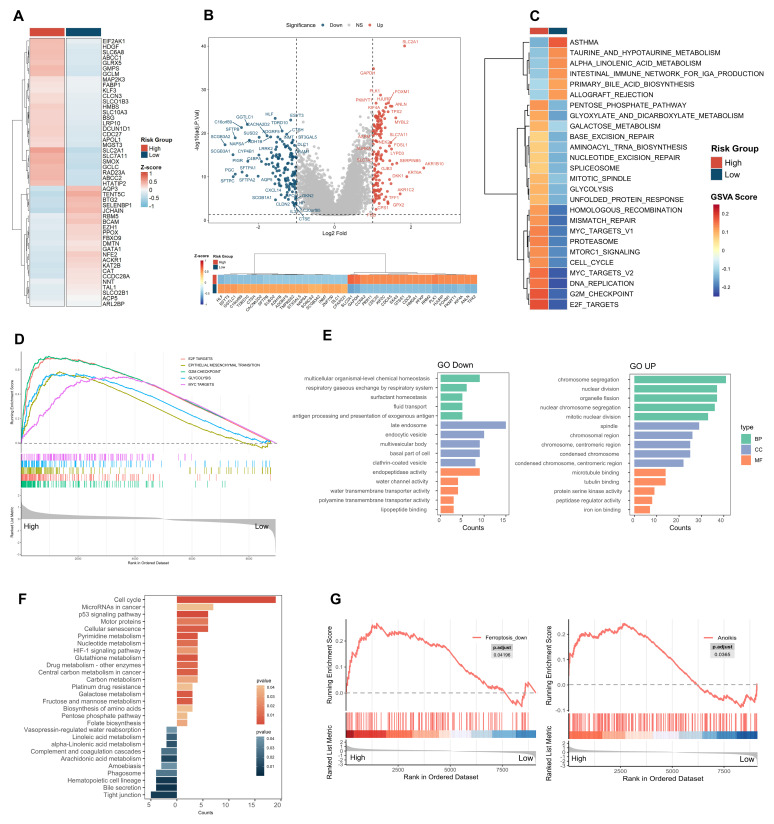
Metabolic reprogramming and ferroptosis regulation are key differences in HMRS-based groups. (**A**) Expression characteristics of 49 HMGs across HMRS-based groups; (**B**) Volcano plot of differential gene expression analysis between high- and low-risk groups, with the corresponding heatmap displaying the expression patterns of these top 20 genes across samples in each risk group. Dashed horizontal and vertical lines indicate the thresholds for statistical significance (*p* < 0.05) and fold change (|log2FC| > 1), respectively; (**C**) Heatmap of GSVA; (**D**) GSEA plot. The gene sets were derived from the hallmark collection in the MsigDB database. The vertical dashed line at 0 indicates the point of zero enrichment score in the ranked gene list; (**E**) Bar plot of GO analysis for differentially expressed genes. BP, biological process; CC, cellular component; MF, molecular function; (**F**) Bar plot of KEGG analysis for differentially expressed genes; (**G**) GSEA plots of cell death mechanisms (*p* < 0.05). The vertical dashed line at 0 indicates the point of zero enrichment score in the ranked gene list.

To explore the expression characteristics of HMGs across high- and low-risk groups, we conducted a comparative analysis (Figure 4A). The results revealed significantly elevated expression levels of genes such as SLC2A1, SLC7A11, SMOX, and ABCC2 in the high-risk group. Differential gene expression analysis by the limma R package, visualized through a volcano plot (Figure 4B), further highlighted the distinct transcriptional profiles between the two groups. The top 20 most significantly differentially expressed genes are prominently displayed, underscoring the marked transcriptional divergence between the high- and low-risk cohorts. We observed that the high-risk group exhibited an elevated expression of genes related to cell cycle regulation and cell division, such as GTSE1, CCNA2, and CDC20, as well as genes involved in glucose metabolism, such as SLC2A1 and GAPDH. In contrast, the low-risk group showed a higher expression of genes associated with lung and airway epithelial cell function, such as SCGB3A2 and SFTPB, suggesting that lung function may be better preserved, potentially contributing to a lower degree of malignancy. Additionally, the upregulation of genes involved in redox metabolism, such as ADH1B and GGTLC1, in the low-risk group indicates that these cells may undergo appropriate redox regulation, thereby maintaining normal cellular function.

To further characterize the biological features of these metabolic risk subtypes, gene set variation analysis (GSVA) revealed the significant enrichment of pathways related to carcinogenesis and metabolism in the high-risk group (Figure 4C). Key metabolic pathways, including the pentose phosphate pathway, glycolysis, and amino acid metabolism, were prominently activated, reflecting metabolic reprogramming essential for tumor cell energy production and biosynthesis. Additionally, pathways driving cell proliferation—such as MYC targets, the cell cycle, mTORC1 signaling, and DNA repair mechanisms—were significantly upregulated, supporting rapid tumor growth and genomic stability. Pathways related to protein degradation, the unfolded protein response, and RNA splicing further highlighted the high-risk group’s adaptability to cellular stress. Gene set enrichment analysis (GSEA) further corroborated these findings, with the top five pathways exhibiting the highest normalized enrichment scores (NESs) being E2F targets, epithelial–mesenchymal transition (EMT), G2M checkpoint, glycolysis, and MYC targets (Figure 4D). These results indicate that the high-risk group is characterized by features associated with increased proliferative activity, metabolic adaptation, and a potential for tumor progression. From the Gene Ontology (GO) enrichment analysis, we observed that the downregulated genes in the high-risk group were significantly enriched in the lipopeptide binding pathway, while iron ion binding and pathways related to cell growth, such as chromosome segregation, were upregulated (Figure 4E). Kyoto Encyclopedia of Genes and Genomes (KEGG) enrichment analysis confirmed the upregulation of pathways linked to cell proliferation (Figure 4F), metabolic reprogramming, and tumor progression, indicating heightened cell division and DNA synthesis activity. Notably, the enrichment of the platinum drug resistance pathway in the high-risk group may indicate a trend toward increased chemoresistance, suggesting potential clinical relevance. In contrast, the low-risk group demonstrated significant enrichment of pathways associated with lipid metabolism and cellular function maintenance. Notably, pathways such as arachidonic acid metabolism, linoleic acid metabolism, and α-linolenic acid metabolism were prominently activated, a potential role for lipid metabolism in modulating inflammation and supporting cellular homeostasis in this group. Additionally, the enrichment of the tight junction pathway, which plays a critical role in maintaining cell barrier function, may reflect a tendency toward more stable physiological regulation in the low-risk group, potentially contributing to the preservation of normal tissue integrity and function. To further investigate the differences in cell death mechanisms between the high- and low-risk groups, we performed GSEA on 18 cell death pathways. Notably, the high-risk group showed a significant enrichment of ferroptosis suppression pathways (Figure 4G), suggesting a potential association between ferroptosis resistance and enhanced tumor cell survival and progression. Consistent with our earlier findings, the low-risk group likely enhances lipid peroxidation and induces ferroptosis, which may be linked to reduced tumor viability. Together, these results highlight a possible role of lipid metabolism in influencing ferroptosis-related processes, which could contribute to the more favorable prognosis observed in the low-risk group. While other cell death pathways, such as anoikis, were also enriched in the high-risk group, our analysis focused on ferroptosis due to its emerging role in tumor progression and therapeutic potential.

### 2.4. Core Heme Metabolism Genes Validation by a Deep Learning Model

**Figure 5:**
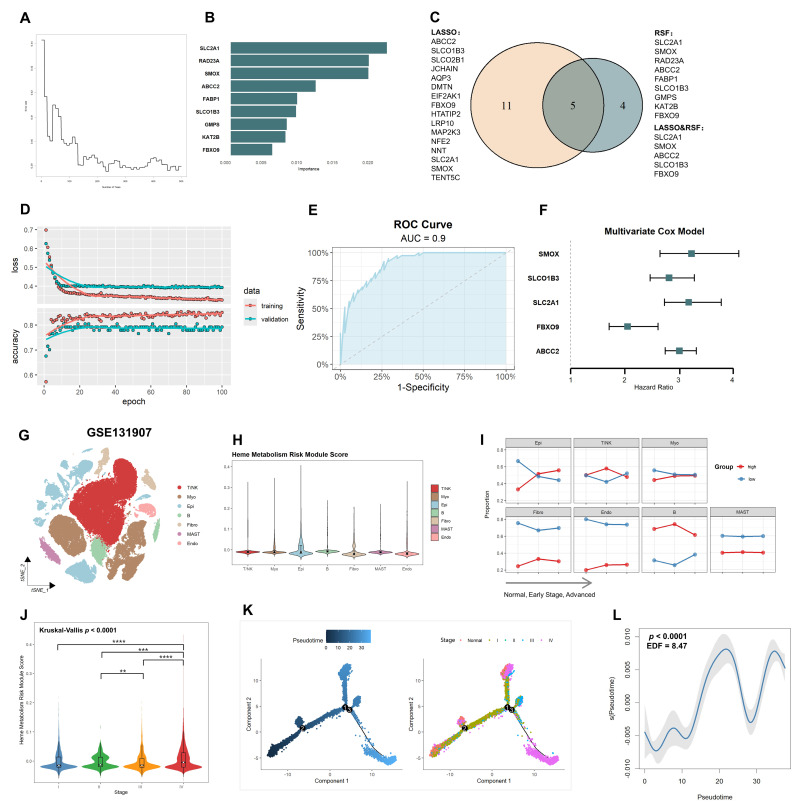
Core heme metabolism genes validation and single-cell level analysis. (**A**) RSF analysis of to identify core heme metabolism genes; (**B**) RSF analysis identified the top 50% of genes contributing most to the model’s predictive ability; (**C**) Venn diagram of gene overlap selected by RSF and LASSO algorithms. The left and right circles represent genes selected by the LASSO and RSF methods, respectively. The overlapping region indicates genes identified by both methods; (**D**) Training and validation performance of the DNN model; (**E**) The ROC curve demonstrates the predictive performance of the model, with an AUC value of 0.9, indicating the strong predictive power of the five gene signature for the distinction of patients into HMRS-based risk groups. The diagonal dashed line represents the performance of a random classifier (AUC = 0.5), serving as a reference baseline; (**F**) Multivariate Cox regression analysis of five core HMGs; (**G**) T-SNE visualization of cell types identified by marker genes; (**H**) Violin plot of heme metabolism risk module score distribution across cell type; (**I**) Line plot illustrating changes in heme metabolism risk module score across cell types during tumor progression; (**J**) Violin plot of heme metabolism risk module score across tumor stages. Overall group differences were assessed using the Kruskal–Wallis test, while pairwise comparisons between individual stages were conducted using the Wilcoxon rank-sum test. ** *p* < 0.01, *** *p* < 0.001, **** *p* < 0.0001; (**K**) Pseudotime trajectory of epithelial cells colored by pseudotime and clinical tumor stage; (**L**) Generalized additive model (GAM) fitting of gene expression dynamics along pseudotime.

**Figure A4:**
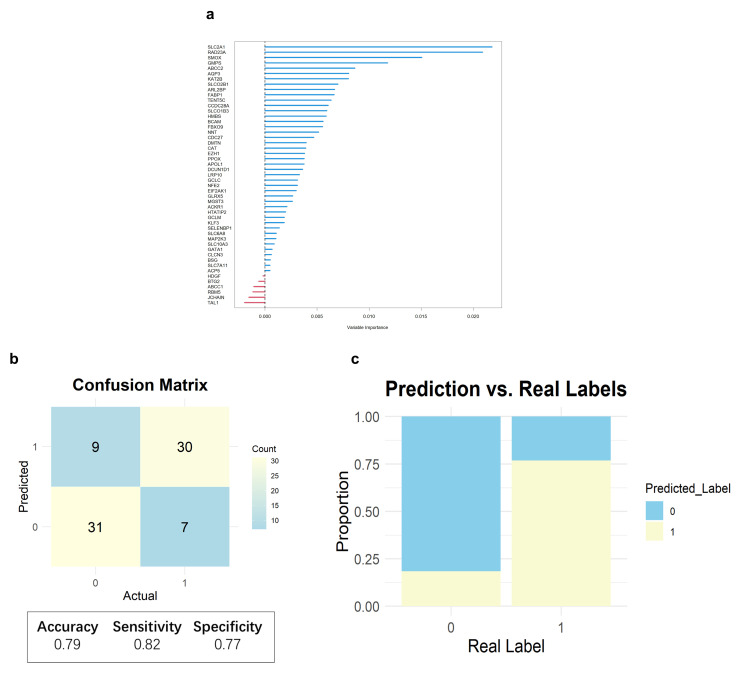
(**a**) Importance ranking of 49 HMGs based on RSF analysis; (**b**) Confusion matrix and performance metrics of model validation; (**c**) Comparison of predicted values and real labels.

In order to further elucidate the characteristics of heme metabolism associated with LUAD prognosis, we utilized random survival forest (RSF) analysis to identify core heme metabolism genes closely linked to patient outcomes (Figure 5A). By calculating the cumulative average importance value of each gene and selecting those with a cumulative proportion of ≤0.5, we identified the subset of genes that contribute the most to the model’s predictive ability (Figure 4B and Figure A4a). Integrating the screening results from the RSF and LASSO algorithms, we identified SLC2A1, SMOX, ABCC2, SLCO1B3, and FBXO9 as the core genes of heme metabolism risk (Figure 5C).

To validate the predictive capability of the five heme metabolism genes, we developed a deep neural network (DNN) classification model incorporating an attention mechanism. The model was trained using the TCGA-LUAD cohort, with the expression levels of the five heme metabolism genes as the input and the HMRS risk group as the classification label (Figure 5D). During validation, the model demonstrated robust performance, achieving an accuracy of 0.79, a sensitivity of 0.82, a specificity of 0.77, and an AUC of 0.90 (Figure 5E and Figure A4b,c). This result further underscores the critical role of core genes related to heme metabolism risk in the classification of populations based on heme metabolism risk and the prognostic assessment of LUAD. Multivariate Cox regression analysis demonstrated that the predictive model constructed from these genes was significantly associated with an increased risk of patient mortality, highlighting the clinical relevance and utility of the model (Figure 5F).

### 2.5. Single-Cell Level Reveals Elevated Risk of Heme Metabolism in Epithelial Cells Driving Tumor Progression

**Figure A5:**
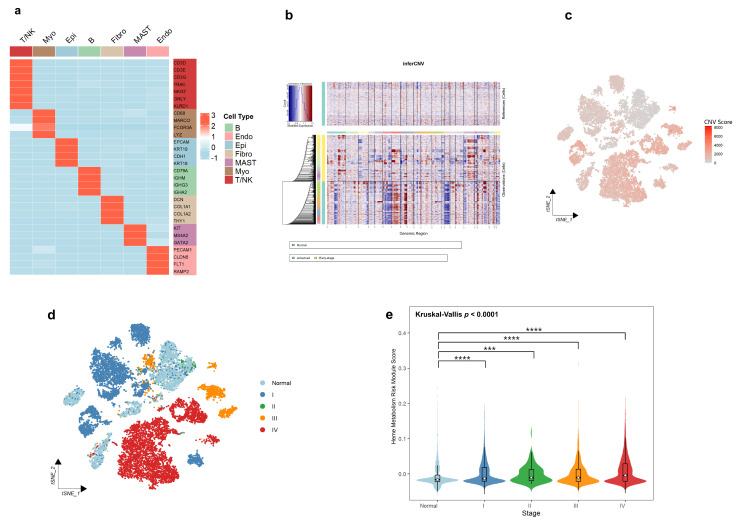
(**a**) Heatmap of cell types and marker gene expression in single-cell RNA sequencing data; (**b**) CNV heatmap of epithelial cells inferred using inferCNV; (**c**) T-SNE visualization of CNV Score; (**d**) T-SNE visualization of tumor stages; (**e**) Violin plot of heme metabolism risk module score across normal and tumor stages. Overall group differences were assessed using the Kruskal–Wallis test, while pairwise comparisons between individual stages were conducted using the Wilcoxon rank-sum test. *** *p* < 0.001, **** *p* < 0.0001.

We used GSE131907 single-cell RNA sequencing data and annotated it with marker genes of different cell types to classify cells into seven major clusters, including T/NK cells, namely, myeloid cells, epithelial cells, B cells, fibroblasts, mast cells, and endothelial cells (Figure 5G and Figure A5a). To quantify the heme metabolism risk in different cell types, we used the “AddModuleScore” function in the Seurat package to calculate the expression levels of five core genes of heme metabolism risk in all cell types (Figure 5H). Among the seven distinct cell types, the risk score of epithelial cells was found to be significantly higher than that of the other types. Further analysis of all cells grouped according to heme metabolism score demonstrated that the proportion of cells with a high heme metabolism score in epithelial cells gradually increased with tumor progression (Figure 5I). Based on CNV profiles, epithelial cells were further stratified into tumor and normal subgroups (Figure 5J and Figure A5b–d). A stage-dependent rise in the proportion of tumor epithelial cells with high heme metabolism scores was evident from normal tissue through to stage IV (Figure 5J and Figure A5e), suggesting a potential association between heme metabolic activity and tumor advancement. To capture the dynamic nature of this process, we applied a generalized additive model (GAM) to examine the relationship between pseudotime and heme metabolism risk module scores (Figure 5L). Pseudotime was used as a proxy for tumor progression (Figure 5K). The GAM curve (EDF = 8.47) revealed a complex, non-linear pattern. Overall, heme metabolism risk module scores increased with pseudotime, but sharp local declines were also observed. These fluctuations may reflect intratumoral heterogeneity and differences in epithelial cell states.

### 2.6. HMRS Panel as a Predictive Biomarker for Chemotherapy Sensitivity

**Figure 6:**
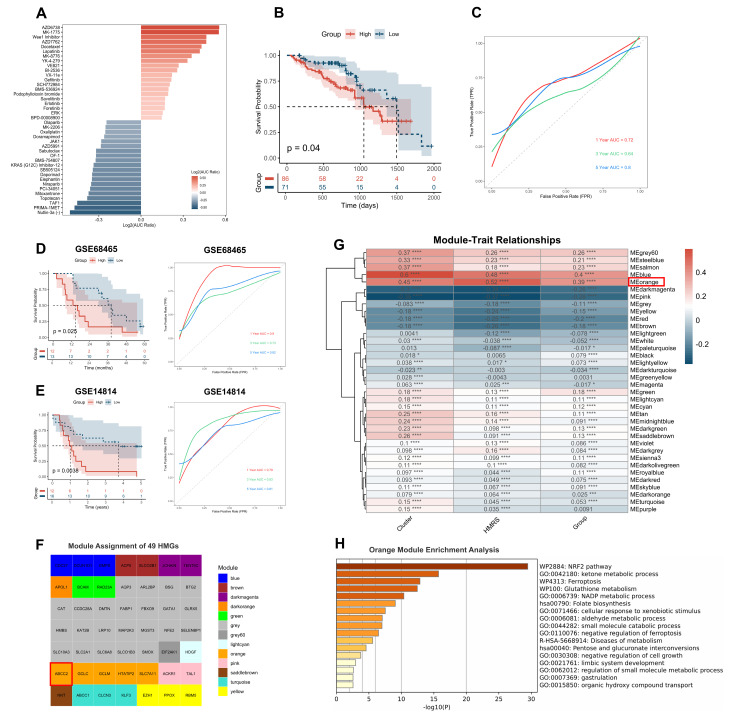
HMRS Panel as a predictive biomarker for chemotherapy sensitivity and identification of ABCC2 as the core gene by WGCNA. (**A**) Bar plot of drug sensitivity analysis revealing distinct patterns between HMRS-based risk groups; (**B**) Kaplan–Meier survival analysis distinguished the prognosis of TCGA-LUAD patients treated with platinum-based chemotherapy based on the HMRS panel; (**C**) Time-dependent ROC curve analysis of TCGA-LUAD patients treated with platinum-based chemotherapy; (**D**) Kaplan–Meier survival analysis and time-dependent ROC curve analysis of GSE68465 validation cohort; (**E**) Kaplan–Meier survival analysis and time-dependent ROC curve analysis of GSE14814 validation cohort; (**F**) module assignment of 49 HMGs identified by WGCNA; (**G**) Module–trait correlation analysis. * *p* < 0.05, ** *p* < 0.01, *** *p* < 0.001, **** *p* < 0.0001; (**H**) Functional enrichment analysis of genes in the orange module.

**Figure A6:**
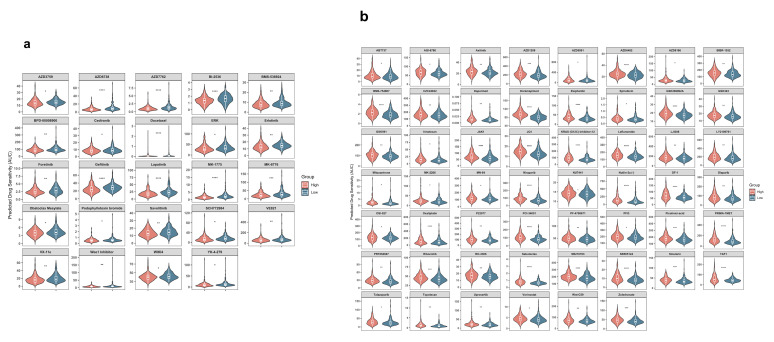
(**a**) Violin plot showing higher drug sensitivity in the high-risk group (*p* < 0.05); (**b**) Violin plot showing higher drug sensitivity in the low-risk group (*p* < 0.05). * *p* < 0.05, ** *p* < 0.01, *** *p* < 0.001, **** *p* < 0.0001.

To investigate the differences in sensitivity to conventional chemotherapeutic drugs between the two risk groups, we analyzed data from the GDSC2 database. In the TCGA-LUAD cohort, drug sensitivity analysis revealed distinct patterns between the HMRS groups (Figure 6A and Figure A6a,b). Patients in the high-risk group showed increased sensitivity to paclitaxel but significantly reduced sensitivity to oxaliplatin compared to the low-risk group. Additionally, the high-risk group exhibited lower sensitivity to drugs targeting key signaling pathways (e.g., PI3K and JAK/STAT inhibitors), cell cycle regulation (e.g., CDK4/6 inhibitors), DNA damage repair (e.g., PARP inhibitors), and the restoration of mutant p53 function (e.g., Nutlin-3a). These findings suggest that the high-risk group may develop resistance to multiple therapeutic agents, emphasizing the challenges in treating these patients and highlighting the importance of personalized therapeutic strategies based on the HMRS panel.

Building on these observations, we further evaluated the predictive ability of the HMRS panel for chemotherapy outcomes in LUAD patients. Chemotherapeutic drugs used in LUAD primarily inhibit tumor cell proliferation through mechanisms such as DNA damage [20], cell cycle disruption [21], and topoisomerase inhibition [22]. These drugs are often administered in combination chemotherapy regimens or alongside other treatments, such as targeted therapies [23] and immunotherapies [24], to enhance efficacy. To assess the clinical relevance of HMRS, we analyzed TCGA cohort patients who received platinum-based chemotherapy. Kaplan–Meier analysis revealed that patients in the high HMRS group had significantly worse prognoses compared to those in the low HMRS group (*p* < 0.05) (Figure 6B). ROC curve analysis demonstrated robust predictive performance, with AUC values of 0.72, 0.64, and 0.80 at 1, 3, and 5 years, respectively (Figure 6C). Validation in the GSE68465 cohort yielded AUCs of 0.90, 0.73, and 0.82 (Figure 6D), while the GSE14814 cohort showed AUCs of 0.79, 0.83, and 0.82 (Figure 6E) for the same time points. These results underscore the strong predictive performance of the HMRS panel in evaluating chemotherapy efficacy, further supporting its clinical utility in guiding treatment decisions for LUAD patients.

### 2.7. ABCC2 Is the Core Gene Identified by WGCNA

**Figure A7:**
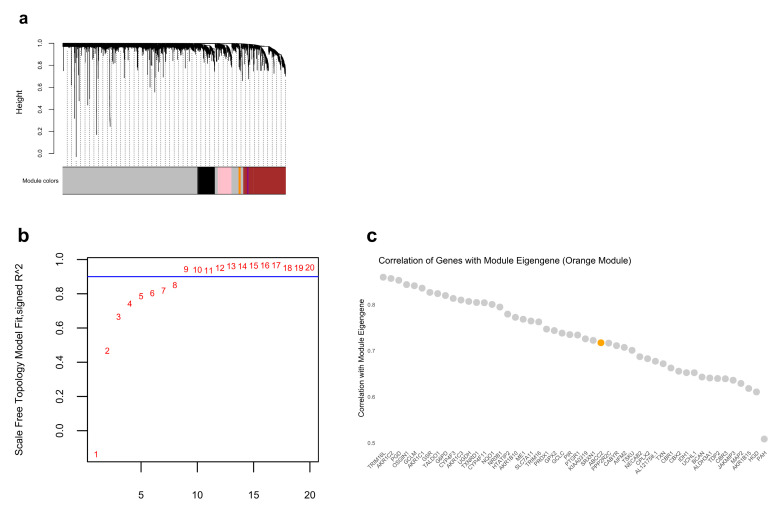
(**a**) Hierarchical clustering dendrogram in WGCNA analysis. Different colors below the dendrogram represent distinct gene co-expression modules identified by hierarchical clustering; (**b**) Determination of the soft-thresholding power in WGCNA analysis. The y-axis shows the scale-free topology fit index (signed R²), and the x-axis represents the soft-thresholding power values from 1 to 20. Red numbers indicate the corresponding power values. The blue horizontal line denotes the threshold R² = 0.90. Power 8 was selected as the soft-thresholding power, as it is the lowest value at which the scale-free topology fit index reaches the threshold of R² = 0.90; (**c**) Gene contribution analysis of the orange module.

To explore highly correlated genes within HMGs, we performed weighted gene co-expression network analysis (WGCNA) using the TCGA-LUAD cohort to identify gene modules and core genes. Initially, we set the soft threshold power to eight and constructed a weighted gene co-expression network using genome-wide expression data from 405 samples with assigned HMRS, encompassing 19,938 genes. Following standard WGCNA procedures, a gene dendrogram was generated and 36 distinct co-expression modules were identified, each represented by a different color (module colors) (Figure A7a,b). These modules reflect groups of genes sharing similar expression patterns across samples. Subsequently, we mapped the 49 HMGs onto the pre-defined modules to assess their distribution. These genes were found to be distributed across 13 modules, reflecting the biological diversity and complex regulatory characteristics of heme metabolism rather than indicating artificial fragmentation. (Figure 6F). Notably, four of the core genes were assigned to the gray module, which represents genes that are not co-expressed with any defined module and are therefore typically excluded from downstream network-based analyses, while ABCC2 was assigned to the orange module. A subsequent analysis of module–clinical trait correlations (including cluster, HMRS, and HMRS group) demonstrated that the orange module was strongly associated with these three clinical traits (Figure 6G).

To further investigate the biological significance of the orange module, we extracted all genes within this module and performed functional enrichment analysis (Figure 6H). The results revealed that the enriched pathways were closely related to redox metabolism. Importantly, GO analysis highlighted pathways associated with the negative regulation of ferroptosis, aligning with our previous findings. In the gene contribution analysis, ABCC2 exhibited a relatively high contribution score (0.72) to the orange module, suggesting its potential involvement in heme metabolism and ferroptosis (Figure A7c). These findings suggest that ABCC2 may play an important role within the HMRS panel, potentially linking heme metabolism with ferroptosis suppression. Its involvement in these processes points to its possible relevance as a therapeutic target and supports the need for further investigation into its role in tumor progression and treatment resistance. 

### 2.8. Inhibition of ABCC2 Significantly Promotes Cisplatin-Induced Ferroptosis

**Figure 7:**
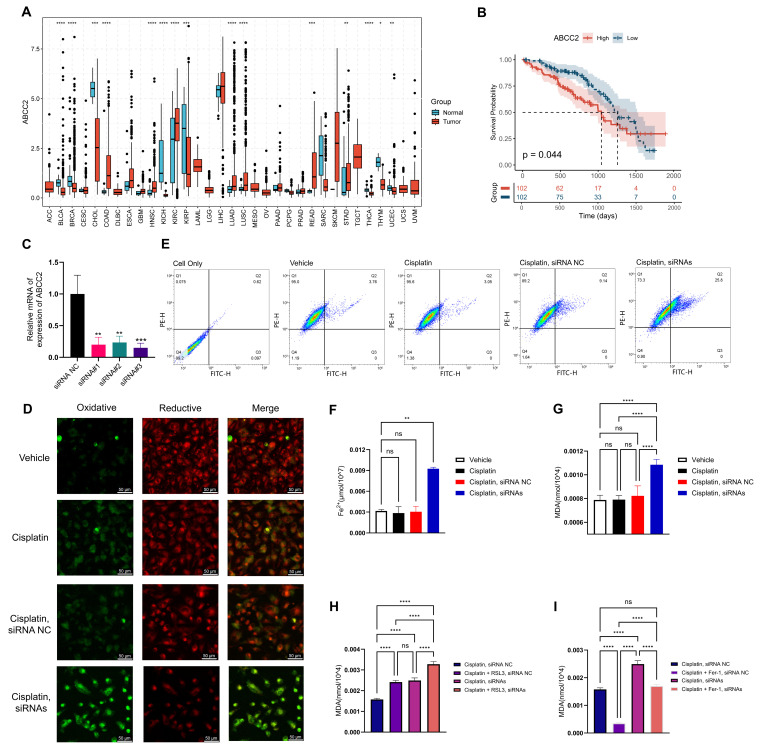
Inhibition of ABCC2 significantly promotes cisplatin-induced ferroptosis. (**A**) Expression patterns of ABCC2 across tumor and normal tissues in the TCGA pan-cancer dataset. Statistical significance is indicated as follows: ns = not significant, * *p* < 0.05, ** *p* < 0.01, *** *p* < 0.001, **** *p* < 0.0001; (**B**) Kaplan–Meier survival analysis stratified by ABCC2 expression levels (**C**) Validation of ABCC2 knockdown efficiency using three independent siRNAs; (**D**) Confocal microscopy images showing lipid peroxidation levels in cisplatin-resistant A549 cells and ABCC2 siRNA-treated cisplatin-resistant A549 cells. Green fluorescence indicates oxidized lipids, while red fluorescence represents non-oxidized lipids. Merged images show the overlay of oxidative and reductive signals. Scale bar: 50 µm; (**E**) Flow cytometry analysis of lipid peroxidation in cisplatin-resistant A549 cells and ABCC2 siRNA-treated cisplatin-resistant A549 cells. Horizontal and vertical lines indicate gating boundaries used to define cell subpopulations. Color intensity reflects cell density, with red indicating higher density and blue indicating lower density; (**F**) ABCC2 inhibition enhances Fe^2+^ ion accumulation in cisplatin-resistant A549 cells; (**G**) ABCC2 inhibition promotes MDA content in cisplatin-resistant A549 cells; (**H**) Promotion of MDA accumulation by the positive ferroptosis inducer RSL3; (**I**) Reduction of MDA levels by negative ferroptosis inhibitor Fer-1.

We evaluated the expression patterns of ABCC2 across tumor and normal tissues using the TCGA pan-cancer dataset (Figure 7A). The analysis revealed significant variations in ABCC2 expression between tumor and adjacent normal tissues across multiple cancer types. Specifically, in LUAD, ABCC2 expression was significantly elevated in tumor tissues compared to normal tissues, suggesting its potential involvement in LUAD pathogenesis and progression. To further explore the clinical relevance of ABCC2, we analyzed its expression in relation to clinical outcomes in TCGA-LUAD cohort patients. By stratifying patients into high- and low-expression groups based on the quartile of ABCC2 expression, Kaplan–Meier analysis demonstrated that patients with high ABCC2 expression had significantly worse prognoses compared to those with low expression (*p* < 0.05) (Figure 7B).

Cisplatin has been shown to induce ferroptosis in certain cell types, underscoring the potential of ferroptosis induction as a therapeutic strategy to combat cisplatin resistance [25,26]. ABCC2, a well-known multidrug resistance protein, primarily mediates chemoresistance through drug efflux [27]. However, whether ABCC2 can modulate chemosensitivity by regulating ferroptosis remains unexplored in LUAD. To investigate this, we performed a series of experiments using cisplatin-resistant A549 cells. Three independent siRNAs targeting ABCC2 were used, all of which effectively knocked down ABCC2 expression (Figure 7C). Using BODIPY 581/591 C11 as a lipid peroxidation sensor, confocal microscopy demonstrated that lipid peroxidation levels in cisplatin-resistant A549 cells were unaltered by 50μM cisplatin treatment. In contrast, cisplatin treatment elicited a significant elevation in lipid oxidation states in cells treated with ABCC2 siRNA relative to the control group (Figure 7D). This observation was further corroborated by flow cytometry analysis (Figure 7E), indicating that ABCC2 inhibition enhances cisplatin-induced lipid peroxidation. Mechanistically, ABCC2 inhibition elevated intracellular lipid peroxidation levels, significantly increased Fe^2+^ ion accumulation (*p* < 0.01) (Figure 7F), and promoted malondialdehyde (MDA) content (*p* < 0.0001) (Figure 7G). These findings suggest that ABCC2 modulates tumor cell sensitivity to cisplatin by regulating the ferroptosis pathway, providing a novel theoretical foundation for overcoming cisplatin resistance in LUAD. Notably, MDA levels in the cisplatin + siABCC2 group were similar to those in the cisplatin + RSL3 group, suggesting that ABCC2 knockdown induces lipid peroxidation comparable to ferroptosis activation. Fer-1 treatment significantly reduced MDA levels in the cisplatin + siABCC2 group to levels close to the cisplatin + NC group, indicating a ferroptosis-dependent effect. Although RSL3 increased MDA levels and Fer-1 reduced them in both NC and siABCC2 groups, the changes were more evident in the siABCC2 group, supporting a role for ABCC2 in regulating ferroptosis sensitivity.

## 3. Discussion

Heme metabolism, a critical component of energy metabolism and redox homeostasis, has been strongly implicated in the pathogenesis and progression of various tissue diseases [17,28]. Elevated hemoglobin uptake is associated with an increased risk of multiple cancers [28]. Notably, non-small-cell lung cancer (NSCLC) cells demonstrate enhanced hemoglobin synthesis and uptake compared to normal lung tissues, resulting in significantly elevated mitochondrial hemoglobin levels, oxygen consumption rate (OCR), and ATP production, thereby promoting tumorigenesis and growth [4]. Our study further underscores the significance of heme metabolism in LUAD. In the chemotherapy cohort, we observed that elevated heme metabolism risk based on HMGs was associated with poorer prognosis, reinforcing its potential role as a prognostic marker and therapeutic target in LUAD.

In this study, we conducted a comprehensive bioinformatics analysis of heme metabolism in LUAD by integrating data from TCGA-LUAD, three GEO RNA-seq cohorts, and one scRNA-seq cohort. A total of 282 heme metabolism-related genes were curated from five authoritative sources, including Reactome, WikiPathways, and MSigDB Hallmark, and validated through published literature [4,15,17,29]. Using LASSO regression, we developed a robust prognostic panel, termed HMRS, which exhibited a high AUC in both the TCGA-LUAD training set and an independent external validation cohort. The HMRS panel effectively stratified LUAD patients into high-risk and low-risk groups, with the high-risk group associated with increased malignant proliferation, elevated tumor metabolism, and reduced ferroptosis activity. To facilitate clinical application, we constructed a nomogram based on the HMRS panel, providing a user-friendly tool for prognostic assessment. Furthermore, we identified five core heme metabolism-related genes (SLC2A1, SMOX, ABCC2, SLCO1B3, and FBXO9) and established a diagnostic model using deep learning algorithms to assess its classification efficiency. Single-cell analysis revealed a significant correlation between elevated heme metabolism risk in epithelial cells and tumor progression, suggesting its potential utility as a biomarker for disease advancement. Through WGCNA, ABCC2 emerged as a key molecule potentially mediating ferroptosis inhibition in heme metabolism subtypes. This finding prompted further cellular-level investigations, which demonstrated that ABCC2 knockdown in cisplatin-resistant A549 cells significantly enhanced cisplatin-induced lipid peroxidation, accompanied by increased intracellular Fe^2+^ levels and elevated MDA content. These results underscore the critical role of ABCC2 in modulating ferroptosis and drug sensitivity, highlighting its potential as a therapeutic target in LUAD. ABCC2, also known as ATP binding cassette subfamily C member 2, encodes multidrug resistance-associated protein 2 (MRP2) [30]. MRP2 is an ABC transporter involved in bilirubin metabolism [31] and drug efflux, including anticancer agents [27]. In non-small-cell lung cancer (NSCLC), elevated ABCC2 expression in cisplatin-resistant A549 cells promotes drug resistance by facilitating drug efflux and inhibiting apoptosis [27]. Although ABCC2 is not traditionally associated with ferroptosis [32], our experiments demonstrate that its inhibition enhances cisplatin-induced ferroptosis, likely through regulating intracellular iron accumulation and lipid metabolism. These findings position ABCC2 as a key regulator of ferroptosis-mediated drug sensitivity, providing novel insights into overcoming cisplatin resistance in NSCLC. Interestingly, another study reported a distinct role of ABCC2 in gastric cancer (GC) [33]. It was found that ABCC2 is upregulated in GC and enhances glutathione (GSH) efflux, inducing metabolic vulnerability and ferroptosis in gastric cancer cells, thereby suppressing tumor growth and improving chemosensitivity [33]. The observed differences in ABCC2’s role may stem from several factors. First, cancer type-specific variations in molecular and metabolic features likely contribute to its divergent functions. Second, the experimental conditions differed as in GC, ABCC2’s effects were studied under amino acid restriction, mimicking metabolic stress, whereas in NSCLC its role was examined in the context of cisplatin treatment. Third, molecular mechanisms may vary, for example, in GC, ABCC2 induces ferroptosis via glutathione metabolism and oxidative stress, while in NSCLC it may influence ferroptosis through iron accumulation and lipid metabolism.

While analyzing drug sensitivities in different HMRS risk groups using the GDSC2 database, we observed a notable pattern: the high-risk group exhibited greater sensitivity to EGFR inhibitors (e.g., Gefitinib, Lapatinib, and AZD3759), whereas the low-risk group showed higher sensitivity to KRAS inhibitors. In NSCLC, approximately 50% of non-squamous patients harbor oncogenic driver mutations, which influence drug response and therapeutic outcomes [34]. This finding suggests that heme metabolism alterations may be closely linked to specific oncogenic mutation profiles, highlighting a potential interplay between heme metabolism and oncogenic signaling pathways that warrants further investigation.

The heterogeneity of NSCLC and its interaction with the tumor microenvironment pose significant challenges for molecular classification. Although k = 2 provided clearer separation in our heme metabolism-based clustering, the k = 3 classification still showed potential biological relevance. The intermediate cluster identified at k = 3 may reflect a transitional state between low- and high-risk groups, suggesting dynamic changes in metabolic states during tumor progression. This intermediate phenotype warrants further functional investigation. In our single-cell analysis, we focused on tumor versus non-tumor epithelial cells. However, lung tissue contains multiple epithelial subtypes, such as AT1, AT2, and Club cells, which represent distinct differentiation states. Tumor epithelial cells may also include subpopulations with different metabolic profiles and biological roles. Future studies should aim to resolve this cellular diversity to refine risk classification and better understand metabolic heterogeneity in LUAD.

Despite the strengths of our study, several limitations should be noted. (1) The gene set we selected was validated through both literature support and functional enrichment analysis, confirming its strong representation of core heme-related pathways. Nevertheless, due to the complex regulation of heme metabolism and its interaction with iron homeostasis and oxidative stress, some relevant regulatory components may not have been fully captured. This underscores the need for continuous refinement of pathway-based gene sets as our understanding of metabolic networks deepens. (2) While our findings highlight the potential clinical relevance of heme metabolism-based risk stratification in LUAD, the translation of gene expression signatures into routine clinical decision-making remains a major challenge. To address this, we have begun developing a detection strategy based on heme metabolism markers and plan to conduct prospective validation studies to evaluate its predictive value and clinical applicability. These efforts aim to help bridge the gap between molecular profiling and real-world patient management. (3) In our study, we also analyzed the mutation landscape of the prognosis-associated heme metabolism-related genes and found that the mutation frequency of individual genes was relatively low (mostly below 2%), limiting further statistical correlation with clinical features or HMRS subgroups. These findings indicate the need for more comprehensive datasets, particularly those with detailed genomic mutation profiles, to better elucidate the relationship between HMGs alterations and clinical outcomes.

In summary, our study uncovers a previously unrecognized role of heme metabolism in LUAD and highlights its potential clinical significance in guiding personalized treatment strategies. We developed the first population-based molecular panel reflecting heme metabolism activity, which can stratify patients by prognosis and chemotherapy response. Notably, we identified ABCC2 as a core gene within this signature and showed that its knockdown promotes ferroptosis and enhances cisplatin sensitivity in resistant LUAD cells. These findings reveal a novel link between heme metabolism, ferroptosis, and treatment response, offering a new avenue for both mechanistic studies and biomarker development in LUAD.

## 4. Materials and Methods

### 4.1. Data Collection and Processing

Clinical information, transcriptome expression, and copy number variation (CNV) and single nucleotide variation (SNV) data for TCGA-LUAD patients were obtained from the TCGA database. Three GEO RNA-seq datasets of LUAD patients were obtained from the GEO database: GSE31210, GSE68465, and GSE14814. Genes associated with heme metabolism were derived from the molecular signatures database (MSigDB; https://www.gsea-msigdb.org/gsea/msigdb, accessed on 6 May 2025), including gene sets from REACTOME_Heme_Biosynthesis, REACTOME_Heme_Degradation, Wikipathway_Heme_Biosynthesis, REACTOME_Scavenging_Heme_from_Plasma, and HALLMARK_Heme_Metabolism (Table A1). These gene sets were further validated through literature review and functional enrichment analysis to ensure their relevance to heme metabolism. (Table A1). The dataset of 18 cell death pathways was compiled from literature [35], the MSigDB database, and the FerrDb V2 database, http://www.zhounan.org/ferrdb/current/ (accessed on 6 May 2025).

### 4.2. Consensus Clustering

We performed consensus clustering using the ConsensusClusterPlus R package (version 1.70.0) to categorize lung adenocarcinoma (LUAD) patients into two distinct subgroups based on gene expression profiles. The clustering was conducted with a maximum number of clusters set to five. A total of 1000 resampling iterations were performed, with 80% of samples randomly selected in each iteration and all genes included. The k-means clustering algorithm was used, and Euclidean distance was applied to measure similarity between samples. Each sample was then assigned to its corresponding subgroup for downstream analysis.

### 4.3. Construction of the HMRS Panel

To further refine our analysis, we employed a LASSO-regularized Cox proportional hazards model with 100 iterations using the glmnet R package (version 4.1-8). Each iteration was performed with 10-fold cross-validation to identify the optimal penalty parameter (lambda) that minimized the partial likelihood deviance. The model was specified with the Cox family and L1 regularization (alpha set to 1). During model fitting, gene expression values were standardized for cross-validation and unstandardized for the final model. Regression coefficients were extracted for each iteration, and genes not selected in a given iteration were assigned a coefficient of zero. This approach allowed us to determine the frequency of occurrence and regression coefficients of each gene in the risk model construction. Using a scoring metric (score = frequency × contribution), we selected the top 50% of genes that exhibited the highest contribution to the risk model (16 genes: ABCC2, SLCO1B3, SLCO2B1, JCHAIN, AQP3, DMTN, EIF2AK1, FBXO9, HTATIP2, LRP10, MAP2K3, NFE2, NNT, SLC2A1, SMOX, and TENT5C). Based on these results, we developed the heme metabolism risk score (HMRS) panel, defined by the following formula: HMRS = ABCC2 × 0.0738 + SLCO1B3 × 0.0307 + SLCO2B1 × (−0.0524) + JCHAIN × (−0.0341) + AQP3 × (−0.0289) + DMTN × (−0.0999) + EIF2AK1 × 0.0880 + FBXO9 × (−0.0941) + HTATIP2 × 0.0345 + LRP10 × 0.1718 + MAP2K3 × 0.0365 + NFE2 × (−0.0848) + NNT × (−0.0567) + SLC2A1 × 0.0764 + SMOX × 0.0530 + TENT5C × (−0.0631).

### 4.4. Decision Curve Analysis for Evaluating the HMRS Panel

To evaluate the clinical utility of predictive models, we performed decision curve analysis (DCA) using the rmda R package (version 1.6). Logistic regression models were constructed to predict event survival using the following predictors: (1) age, (2) risk score, (3) total score (nomogram), (4) gender, and (5) T stage. Each predictor was included as a separate model, and the logistic regression models were fitted with a binomial family and logit link. Decision curves were generated for each model, with threshold probabilities ranging from 0 to 1 (in increments of 0.01). The net benefit of each model was calculated and plotted against the threshold probability to assess the clinical utility of the predictors.

### 4.5. Comparative Analysis of Cluster-Based Subtyping and HMRS Risk Groups

To evaluate the consistency and similarity between a Cluster and an HMRS Group, we generated a confusion matrix to compare the distribution of samples across Cluster and Group categories. To quantify the similarity between Cluster and Group, we converted both categorical variables into dummy matrices and computed the Jaccard similarity index using the vegan package (version 2.6-10). The Jaccard index, ranging from 0 (no similarity) to 1 (complete similarity), was used to measure the overlap between the two classification schemes. This integrated approach allowed us to systematically evaluate the alignment between cluster-derived subgroups (unsupervised clustering) and predefined HMRS groups (supervised stratification), providing insights into the robustness and biological relevance of the classification methods.

### 4.6. Core Heme Metabolism Genes Identification

To identify core heme metabolism-related genes, we employed a random survival forest (RSF) model implemented in the randomForestSRC R package (version 3.3.3). The model was trained using survival data, with the optimal number of trees determined by evaluating the out-of-bag (OOB) error rate across 1 to 500 trees, selecting the number of trees corresponding to the lowest error rate. Feature importance scores were calculated for each gene, and genes with importance scores greater than 0.5 were considered significant. To ensure robustness, the RSF model was iterated 100 times with different random seeds, and the average importance scores across all iterations were used to rank the genes. The top 50% of genes based on cumulative importance scores were selected for further analysis. These genes were then intersected with those identified by LASSO regression, resulting in the identification of 5 core heme metabolism-related genes.

### 4.7. Deep Learning Model Construction

To validate the discriminative power of the 5 core heme metabolism-related genes in stratifying LUAD patients by risk, we constructed a deep neural network (DNN) model using the keras R package (version 2.15.0) with TensorFlow (version 2.16.0) as the backend. The TCGA-LUAD dataset was preprocessed to retain the core genes and relevant clinical variables, and it was randomly split into training (80%) and test (20%) sets using the caret package (version 7.0-1). The DNN architecture consisted of an input layer (64 neurons, ReLU activation), a hidden layer (32 neurons, ReLU activation), and an output layer (1 neuron, sigmoid activation). The model was compiled with binary cross-entropy loss, the Adam optimizer, and accuracy as the evaluation metric. Training was performed over 100 epochs with a batch size of 32, and validation was conducted on the test set. Model performance was assessed using accuracy, sensitivity, specificity, and the area under the ROC curve (AUC). Additionally, the training process was visualized through loss and accuracy curves and the discriminative power of the model was further illustrated using a confusion matrix, a bar plot of predicted versus actual labels, and a ROC curve with AUC value.

### 4.8. Functional Enrichment Analysis

To explore biological differences between HMRS-defined groups, we performed gene set variation analysis (GSVA) using the GSVA R package (version 1.50.5). GSVA was conducted with the non-parametric “gsva” method, using a Gaussian kernel density estimation. Gene sets were obtained from MSigDB, with a minimum and maximum gene set size of 10 and 500, respectively. A linear model was then fitted using the limma R package (version 3.58.1), with HMRS group labels included in the design matrix as a categorical variable. Empirical Bayes moderation was applied to assess differential pathway activity. Gene sets with an adjusted *p*-value < 0.05 and absolute log fold change > 0.2 were considered significant. Subsequent functional enrichment analyses were conducted using Gene Ontology (GO), Kyoto Encyclopedia of Genes and Genomes (KEGG), and gene set enrichment analysis (GSEA) via the clusterProfiler R package (version 4.10.1).

### 4.9. Single-Cell RNA-Seq Analysis Data Collection and Processing

We analyzed 26 samples from the GSE131907 dataset, including 11 lung adenocarcinoma (LUAD) tissues, 11 matched adjacent normal tissues, and 4 endobronchial brushing samples from patients with advanced-stage tumors. Single-cell RNA sequencing data were processed using the Seurat R package (version 5.1.0). To ensure high data quality, we applied the following filtering criteria: cells with fewer than 250 detected genes or fewer than 500 unique molecular identifiers (UMIs) were excluded; cells with a log10 ratio of detected genes per UMI (log10GenesPerUMI) below 0.80 were removed to filter out low-complexity cells; cells with over 20% mitochondrial gene content were excluded to avoid inclusion of dying or stressed cells. Potential doublets were identified and removed based on the co-expression of canonical markers from distinct lineages. In addition, cells with a housekeeping gene UMI sum (ACTB, GAPDH, MALAT1) below 1 were removed to exclude transcriptionally inactive or damaged cells.

Cell clustering was performed using Seurat’s “FindClusters” and “FindNeighbors” functions, with results visualized via t-distributed stochastic neighbor embedding (t-SNE). Cell types were annotated based on characteristic marker genes, and the activity levels of specific gene sets in individual cells were quantified using Seurat’s “AddModuleScore” function.

Copy number variation (CNV) analysis was performed using the infercnv R package (version 1.18.1). The key parameters are as follows: the cutoff was set to 0.1, which is appropriate for 10x genomics data; and reference group names were set as “Normal” to define the baseline for CNV inference. Gene positional information was provided using a gene order file based on GENCODE v27 (hg38). Cells from the Early-stage and Advanced groups whose CNV scores did not exceed the median CNV score of the Normal group were considered normal-like cells within tumor tissues and were excluded from the analysis due to their ambiguous classification.

To investigate the dynamic changes in heme metabolism during tumor progression, we performed pseudotime trajectory analysis using the Monocle R package (version 2.30.1). Risk module scores for heme metabolism were mapped onto the trajectory, and a generalized additive model (GAM) was fitted using the mgcv R package (version 1.9-1) to assess the relationship between pseudotime and risk scores.

### 4.10. Drug Sensitivity Prediction

Drug sensitivity data were obtained from the genomics of drug sensitivity in cancer 2 (GDSC2) database, which includes gene expression profiles and drug response data for a wide range of cancer cell lines. The R package oncoPredict (version 1.2) was used to predict drug sensitivity in LUAD patients based on their gene expression profiles. The training data were preprocessed by exponentiating the drug response values and standardizing the gene expression data. The LUAD patient gene expression data were aligned with the GDSC2 data by retaining common genes and removing low-variance genes (20% threshold). Drug sensitivity predictions were generated using the calcPhenotype function with batch correction (standardize) and power transformation of the phenotype data. The predicted drug sensitivity values were merged with patient grouping information (high- vs. low-risk groups) and analyzed for significant differences. Significant drugs (*p* < 0.05) were further visualized using violin plots and boxplots, and the sensitivity ratios, defined as the ratio of the mean AUC values between the low- and high-risk groups, were calculated to identify drugs with higher sensitivity in specific risk groups.

### 4.11. Weighted Gene Coexpression Network Analysis (WGCNA)

Gene expression data from TCGA-LUAD samples, matched with risk scores, were analyzed using weighted gene co-expression network analysis (WGCNA) implemented in the R package WGCNA (version 1.73). The soft-thresholding power (β) was selected using the pickSoftThreshold function to achieve a scale-free topology fit index (R^2^) > 0.9, with the optimal power determined to be 8. Gene co-expression modules were identified using the blockwiseModules function with a minimum module size of 30 genes and a module merging threshold (mergeCutHeight) of 0.25, allowing modules with eigengene correlation above 0.75 to be merged. The topological overlap matrix (TOM) type was set to “unsigned” to focus on the strength rather than the direction of gene–gene correlations. Module eigengenes, representing the overall expression patterns of each module, were calculated and correlated with classification traits, including cluster, HMRS, and HMRS group. Significant module–trait relationships were visualized using heatmaps, with *p*-values annotated to indicate statistical significance. Hub genes within each module were identified based on their high correlation with module eigengenes. Candidate genes of interest were mapped to their respective modules, and their module assignments were visualized using a color-coded tile plot. Functional enrichment analysis of WGCNA gene modules was performed using Metascape (https://metascape.org/gp/index.html, accessed on 6 May 2025), an online tool for identifying associated biological functions and pathways.

### 4.12. Cell Culture and Transfection

The human lung adenocarcinoma cell line A549 was obtained from the American Type Culture Collection (ATCC, Manassas, VA, USA). Cells were cultured in vitro using RPMI-1640 medium (Gibco, Thermo Fisher Scientific, Waltham, MA, USA) supplemented with 15% fetal bovine serum and 1% penicillin-streptomycin (Gibco, Thermo Fisher Scientific, Waltham, MA, USA). A549 cells were continuously exposed to increasing concentrations of cisplatin over several months. The resistance was confirmed by evaluating the half-maximal inhibitory concentration (IC50) of cisplatin in cisplatin-resistant A549 cells compared to the parental A549 cells using a cell viability assay (CCK-8). Small interfering RNA (siRNA) targeting ABCC2 and a negative control siRNA were purchased from RiboBio (Guangzhou, China). The sequences of the ABCC2-targeting siRNAs were as follows: stB0001370A (st-h-ABCC2_001): AGTGGATGCTCATGTAGGA; stB0001370B (st-h-ABCC2_002): GTACCTACAAGCAATAGGA; stB0001370C (st-h-ABCC2_003): AGACATCTATCTTCTAGAT. For transient transfection, cisplatin-resistant A549 cells were transfected with siRNA using Lipofectamine 2000 (Invitrogen, Thermo Fisher Scientific, Waltham, MA, USA) for 48 h, followed by functional assays. Three validated siRNA sequences targeting ABCC2 were mixed in equal proportions and used as a pooled siRNA for all subsequent experiments. Total RNA was extracted using an RNA extraction kit (TOYOBO, Osaka, Japan) according to the manufacturer’s instructions, and cDNA was synthesized for real-time PCR using SYBR Green qPCR mix (Applied Biosystems, Thermo Fisher Scientific, Waltham, MA, USA).

### 4.13. Cellular Lipid Peroxidation Assay

Lipid peroxidation assay was performed using BODIPY 581/591 C11 (Invitrogen, Thermo Fisher Scientific, Waltham, MA, USA). Cisplatin-resistant A549 cells were inoculated in 6-well plates, and when the cell density reached 70–80%, ABCC2 knockdown and control treatments were carried out, respectively, and the cells of the experimental group and the control group were treated with cisplatin for 24 h. After discarding the medium, cells were washed twice with PBS and incubated with the C11-BODIPY 581/591 probe (final concentration: 5 μM) at 37 °C for 30 min in the dark. Unbound probe was removed by washing twice with PBS. For confocal microscopy, cells were imaged at 488 nm (oxidized, green fluorescence) and 581 nm (reduced, red fluorescence) excitation wavelengths. For flow cytometry, stained cells were trypsinized, resuspended in PBS, and analyzed at 488 nm excitation, detecting green fluorescence (FITC channel, oxidized state) and red fluorescence (PE channel, reduced state). At least 10,000 live cells per sample were collected. The primary readout was the shift from the reduced state (Q1, PE-positive) to the oxidized state (Q2, FITC-positive), reflecting increased lipid peroxidation levels.

### 4.14. Detection of Cellular Fe^2+^ Content

Intracellular Fe^2+^ content was measured using a Fe^2+^ assay kit (Solarbio, Beijing, China). Cisplatin-resistant A549 cells were seeded in 10 cm dishes and treated with cisplatin for 24 h after ABCC2 knockdown or control. Cells were washed twice with PBS, collected, and counted, followed by lysis using the kit-provided extraction buffer and ultrasonication on ice. After centrifugation (10,000× *g*, 10 min, 4 °C), the supernatant was mixed with the Fe^2+^ assay working solution and incubated at 37 °C for 30 min in the dark. Absorbance at 593 nm was measured using a microplate reader, and the Fe^2+^ concentration was calculated based on the kit’s standard curve.

### 4.15. Cellular Malondialdehyde (MDA) Content

The malondialdehyde (MDA) content in cells was determined using a commercial MDA assay kit (Solarbio, Beijing, China) following the manufacturer’s protocol. Briefly, harvested cells were centrifuged, and the pellet was resuspended in extraction buffer at a ratio of 1 mL buffer per 5 × 10^6^ cells. The cell suspension was then sonicated on ice (200 W, 3 s pulses with 10 s intervals, 30 cycles) to lyse the cells. The lysate was centrifuged at 8000× *g* for 10 min at 4 °C, and the supernatant was collected for analysis. For the assay, 0.1 mL of the supernatant was mixed with 0.1 mL of reagent 3 and 0.3 mL MDA detection working solution and incubated at 100 °C for 60 min in a tightly sealed tube to prevent evaporation. After cooling on ice, the mixture was centrifuged at 10,000× *g* for 10 min at 25 °C. The supernatant (500 µL) was transferred to a microquartz cuvette or a 96-well plate, and the absorbance was measured at 532 nm and 600 nm using a spectrophotometer. The MDA concentration was calculated based on the difference in absorbance (ΔA = A532 − A600) and quantified using a standard curve.

### 4.16. Statistical Analysis

Data processing and statistical analyses were performed using R (version 4.3.3) programs. Kaplan–Meier survival curves were plotted by the survminer R package (version 0.5.0) for OS analysis and differences between groups were evaluated using the log-rank test. Time-dependent ROC curves were generated using the timeROC package (version 0.4). Both univariate and multivariate Cox regression analyses were performed using the survival R package (version 3.5-8) to assess the associations between clinical variables and patient survival outcomes. For comparisons between two groups, Wilcoxon rank-sum tests were used. For comparisons among more than two groups, the Kruskal–Wallis test followed by pairwise Wilcoxon post hoc tests was applied. Spearman’s rank correlation was used to evaluate associations between continuous variables. *p*-values were adjusted using the Benjamini–Hochberg (BH) method where applicable, and values less than 0.05 were considered statistically significant.

## Data Availability

The data presented in this study are available in The Cancer Genome Atlas (TCGA, https://www.cancer.gov/tcga, accessed on 6 May 2025). and Gene Expression Omnibus (GEO, https://www.ncbi.nlm.nih.gov/geo/, accessed on 6 May 2025). In GEO database, the reference numbers are GSE31210, GSE68465, GSE14814, and GSE131907.

## Abbreviations

The following abbreviations are used in this manuscript:

HMGs	Heme Metabolism-related Genes
ROS	Reactive Oxygen Species
OS	Overall Survival
CNV	Copy Number Variation
CDF	Cumulative Distribution Function
PCA	Principal Component Analysis
ROC	Receiver Operating Characteristic
LASSO	Least Absolute Shrinkage and Selection Operator
HMRS	Heme Metabolism Risk Score
DCA	Decision Curve Analysis
GSVA	Gene Set Variation Analysis
GSEA	Gene Set Enrichment Analysis
EMT	Epithelial–Mesenchymal Transition
GO	Gene Ontology
KEGG	Kyoto Encyclopedia of Genes and Genomes
NESs	Normalized Enrichment Scores
RSF	Random Survival Forest
DNN	Deep Neural Network
t-SNE	t-Distributed Stochastic Neighbor Embedding
TCGA	The Cancer Genome Atlas
GEO	Gene Expression Omnibus
GDSC	Genomics of Drug Sensitivity in Cancer
LUAD	Lung adenocarcinoma
NSCLC	Non-Small-Cell Lung Cancer
MSigDB	Molecular Signatures Database
AUC	Areas Under the Curve
GC	Gastric Cancer
GSH	Glutathione
GAM	Generalized additive model

## Appendix A

**Table A1:** Heme metabolism-related genes.

Symbol									
*COX10*	*CD163*	*IGKV1-5*	*IGLV3-27*	*BTRC*	*EZH1*	*ICAM4*	*NFE2*	*SELENBP1*	*TRAK2*
*COX15*	*HBA1*	*IGKV1D-12*	*IGLV6-57*	*C3*	*FBXO34*	*IGSF3*	*NFE2L1*	*SIDT2*	*TRIM10*
*ALAS1*	*HBA2*	*IGKV1D-16*	*IGLV7-43*	*CA1*	*FBXO7*	*ISCA1*	*NNT*	*SLC10A3*	*TRIM58*
*FECH*	*HBB*	*IGKV1D-33*	*JCHAIN*	*CA2*	*FBXO9*	*KAT2B*	*NR3C1*	*SLC11A2*	*TSPAN5*
*CPOX*	*HP*	*IGKV1D-39*	*LRP1*	*CAST*	*FN3K*	*KDM7A*	*NUDT4*	*SLC22A4*	*TSPO2*
*ABCG2*	*HPR*	*IGKV2-28*	*ABCB6*	*CAT*	*FOXJ2*	*KEL*	*OPTN*	*SLC25A37*	*TYR*
*UROD*	*HPX*	*IGKV2-30*	*ACKR1*	*CCDC28A*	*FOXO3*	*KHNYN*	*OSBP2*	*SLC25A38*	*UBAC1*
*PPOX*	*IGHA1*	*IGKV2D-28*	*ACP5*	*CCND3*	*FTCD*	*KLF1*	*P4HA2*	*SLC2A1*	*UCP2*
*ALAD*	*IGHA2*	*IGKV2D-30*	*ACSL6*	*CDC27*	*GAPVD1*	*KLF3*	*PC*	*SLC30A1*	*USP15*
*ALAS2*	*IGHV1-2*	*IGKV2D-40*	*ADD1*	*CDR2*	*GATA1*	*LAMP2*	*PDZK1IP1*	*SLC30A10*	*VEZF1*
*FLVCR1*	*IGHV1-46*	*IGKV3-11*	*ADD2*	*CIR1*	*GCLC*	*LMO2*	*PGLS*	*SLC4A1*	*XK*
*ALB*	*IGHV1-69*	*IGKV3-15*	*ADIPOR1*	*CLCN3*	*GCLM*	*LPIN2*	*PICALM*	*SLC66A2*	*XPO7*
*UROS*	*IGHV2-5*	*IGKV3-20*	*AGPAT4*	*CLIC2*	*GDE1*	*LRP10*	*PIGQ*	*SLC6A8*	*YPEL5*
*HMBS*	*IGHV2-70*	*IGKV3D-20*	*AHSP*	*CROCCP2*	*GLRX5*	*MAP2K3*	*PPP2R5B*	*SLC6A9*	
*ABCC2*	*IGHV3-11*	*IGKV4-1*	*ALDH1L1*	*CTNS*	*GMPS*	*MARCHF2*	*PRDX2*	*SLC7A11*	
*BLVRB*	*IGHV3-13*	*IGKV5-2*	*ALDH6A1*	*CTSB*	*GYPA*	*MARCHF8*	*PSMD9*	*SMOX*	
*HMOX1*	*IGHV3-23*	*IGLC2*	*ANK1*	*CTSE*	*GYPB*	*MARK3*	*RAD23A*	*SNCA*	
*ABCC1*	*IGHV3-30*	*IGLC3*	*AQP3*	*DAAM1*	*GYPC*	*MBOAT2*	*RANBP10*	*SPTA1*	
*HMOX2*	*IGHV3-33*	*IGLV1-40*	*ARHGEF12*	*DCAF10*	*GYPE*	*MFHAS1*	*RAP1GAP*	*SPTB*	
*BLVRA*	*IGHV3-48*	*IGLV1-44*	*ARL2BP*	*DCAF11*	*H1-0*	*MGST3*	*RBM38*	*SYNJ1*	
*SLCO1B3*	*IGHV3-53*	*IGLV1-47*	*ASNS*	*DCUN1D1*	*H4C3*	*MINPP1*	*RBM5*	*TAL1*	
*SLCO1B1*	*IGHV3-7*	*IGLV1-51*	*ATG4A*	*DMTN*	*HAGH*	*MKRN1*	*RCL1*	*TCEA1*	
*SLCO2B1*	*IGHV4-34*	*IGLV2-11*	*ATP6V0A1*	*E2F2*	*HBBP1*	*MOCOS*	*RHAG*	*TENT5C*	
*FABP1*	*IGHV4-39*	*IGLV2-14*	*BACH1*	*EIF2AK1*	*HBD*	*MOSPD1*	*RHCE*	*TFDP2*	
*UGT1A1*	*IGHV4-59*	*IGLV2-23*	*BCAM*	*ELL2*	*HBQ1*	*MPP1*	*RHD*	*TFRC*	
*GSTA1*	*IGKV1-12*	*IGLV2-8*	*BMP2K*	*ENDOD1*	*HBZ*	*MXI1*	*RIOK3*	*TMCC2*	
*UGT1A4*	*IGKV1-16*	*IGLV3-1*	*BNIP3L*	*EPB41*	*HDGF*	*MYL4*	*RNF123*	*TMEM9B*	
*AMBP*	*IGKV1-17*	*IGLV3-19*	*BPGM*	*EPB42*	*HEBP1*	*NARF*	*RNF19A*	*TNRC6B*	
*APOA1*	*IGKV1-33*	*IGLV3-21*	*BSG*	*EPOR*	*HTATIP2*	*NCOA4*	*SDCBP*	*TNS1*	
*APOL1*	*IGKV1-39*	*IGLV3-25*	*BTG2*	*ERMAP*	*HTRA2*	*NEK7*	*SEC14L1*	*TOP1*	

**Table A2:** 49 prognostic genes associated with OS by univariate Cox regression analysis.

*Gene*	HR	Coef	*p* Value	LowerCI	UpperCI
*PPOX*	0.752504	−0.284348	0.028362	0.58358	0.970326
*HMBS*	1.379057	0.3214	0.009022	1.083454	1.75531
*ABCC2*	1.160223	0.148612	0.000087	1.077248	1.249589
*ABCC1*	1.218173	0.197352	0.043659	1.005626	1.475644
*SLCO1B3*	1.15296	0.142333	0.026213	1.016984	1.307117
*SLCO2B1*	0.873744	−0.134968	0.043410	0.766482	0.996015
*FABP1*	1.407514	0.341825	0.006982	1.097981	1.804308
*APOL1*	1.189261	0.173332	0.019232	1.028612	1.375
*JCHAIN*	0.870827	−0.138312	0.002853	0.795181	0.95367
*ACKR1*	0.885668	−0.121414	0.021099	0.798835	0.981939
*ACP5*	0.825942	−0.19123	0.026820	0.697319	0.978291
*AQP3*	0.883456	−0.123914	0.005288	0.809776	0.96384
*ARL2BP*	1.583942	0.459917	0.022251	1.067797	2.349579
*BCAM*	0.867207	−0.142478	0.027419	0.76407	0.984265
*BSG*	1.328363	0.283948	0.040976	1.011707	1.74413
*BTG2*	0.80163	−0.221108	0.002304	0.695385	0.924108
*CAT*	0.76955	−0.261949	0.010607	0.629476	0.940794
*CCDC28A*	0.701313	−0.354801	0.011501	0.532601	0.923467
*CDC27*	1.516675	0.416521	0.012826	1.092501	2.10554
*CLCN3*	1.362124	0.309045	0.014418	1.063403	1.744758
*DCUN1D1*	1.469944	0.385224	0.005040	1.122989	1.924093
*DMTN*	0.835953	−0.179183	0.020993	0.717961	0.973337
*EIF2AK1*	1.571036	0.451735	0.003926	1.155738	2.135566
*EZH1*	0.753368	−0.283201	0.026424	0.586699	0.967384
*FBXO9*	0.744254	−0.295373	0.026388	0.573441	0.965947
*GATA1*	0.475254	−0.743907	0.006836	0.277209	0.814786
*GCLC*	1.146746	0.136928	0.007932	1.036491	1.268728
*GCLM*	1.176157	0.162253	0.037816	1.00917	1.370776
*GLRX5*	1.553487	0.440502	0.007061	1.127513	2.140394
*GMPS*	1.528959	0.424587	0.000510	1.203396	1.942598
*HDGF*	1.318635	0.276597	0.031325	1.025095	1.696231
*HTATIP2*	1.520409	0.418979	0.000264	1.21393	1.904264
*KAT2B*	0.754193	−0.282107	0.011658	0.60573	0.939044
*KLF3*	1.30228	0.264116	0.048514	1.00172	1.693021
*LRP10*	1.368367	0.313618	0.010557	1.075973	1.740218
*MAP2K3*	1.342451	0.294497	0.039175	1.014722	1.776028
*MGST3*	1.372603	0.316709	0.036875	1.019481	1.848037
*NFE2*	0.842092	−0.171867	0.028823	0.721826	0.982395
*NNT*	0.799911	−0.223255	0.043716	0.643894	0.993731
*RAD23A*	1.833232	0.606081	0.001152	1.272041	2.642006
*RBM5*	0.743686	−0.296136	0.016172	0.584221	0.946678
*SELENBP1*	0.873031	−0.135784	0.013918	0.78349	0.972806
*SLC10A3*	1.348278	0.298829	0.042569	1.010058	1.799754
*SLC2A1*	1.29581	0.259136	0.000020	1.150278	1.459753
*SLC6A8*	1.165467	0.153122	0.024519	1.019865	1.331856
*SLC7A11*	1.123212	0.116192	0.019410	1.018945	1.238148
*SMOX*	1.369945	0.314771	0.000072	1.172704	1.600361
*TAL1*	0.715952	−0.334143	0.043638	0.517495	0.990514
*TENT5C*	0.7314	−0.312794	0.000116	0.62387	0.857464

## References

[1] Bray F., Laversanne M., Sung H., Ferlay J., Siegel R.L., Soerjomataram I., Jemal A. (2024). Global cancer statistics 2022: GLOBOCAN estimates of incidence and mortality worldwide for 36 cancers in 185 countries. CA Cancer J. Clin..

[2] Leiter A., Veluswamy R.R., Wisnivesky J.P. (2023). The Global Burden of Lung Cancer: Current Status and Future Trends. Nat. Rev. Clin. Oncol..

[3] Hanahan D., Weinberg R.A. (2011). Hallmarks of Cancer: The next Generation. Cell.

[4] Sohoni S., Ghosh P., Wang T., Kalainayakan S.P., Vidal C., Dey S., Konduri P.C., Zhang L. (2019). Elevated Heme Synthesis and Uptake Underpin Intensified Oxidative Metabolism and Tumorigenic Functions in Non-Small Cell Lung Cancer Cells. Cancer Res..

[5] Martínez-Reyes I., Chandel N.S. (2021). Cancer Metabolism: Looking Forward. Nat. Rev. Cancer.

[6] Cai X., Miao J., Sun R., Wang S., Molina-Vila M.A., Chaib I., Rosell R., Cao P. (2021). Dihydroartemisinin Overcomes the Resistance to Osimertinib in EGFR-Mutant Non-Small-Cell Lung Cancer. Pharmacol. Res..

[7] Suresh S., Chen B., Zhu J., Golden R.J., Lu C., Evers B.M., Novaresi N., Smith B., Zhan X., Schmid V. (2020). eIF5B Drives Integrated Stress Response-Dependent Translation of PD-L1 in Lung Cancer. Nat. Cancer.

[8] Torres Á., Quintanilla F., Barnafi E., Sánchez C., Acevedo F., Walbaum B., Merino T. (2024). Dietary Interventions for Cancer Prevention: An Update to ACS International Guidelines. Nutrients.

[9] Tasevska N., Sinha R., Kipnis V., Subar A.F., Leitzmann M.F., Hollenbeck A.R., Caporaso N.E., Schatzkin A., Cross A.J. (2009). A Prospective Study of Meat, Cooking Methods, Meat Mutagens, Heme Iron, and Lung Cancer Risks. Am. J. Clin. Nutr..

[10] Zhang C., Liu X., Jin S., Chen Y., Guo R. (2022). Ferroptosis in Cancer Therapy: A Novel Approach to Reversing Drug Resistance. Mol. Cancer.

[11] Stockwell B.R. (2022). Ferroptosis Turns 10: Emerging Mechanisms, Physiological Functions, and Therapeutic Applications. Cell.

[12] Dixon S.J., Lemberg K.M., Lamprecht M.R., Skouta R., Zaitsev E.M., Gleason C.E., Patel D.N., Bauer A.J., Cantley A.M., Yang W.S. (2012). Ferroptosis: An Iron-Dependent Form of Nonapoptotic Cell Death. Cell.

[13] Wang Y., Wu X., Ren Z., Li Y., Zou W., Chen J., Wang H. (2023). Overcoming Cancer Chemotherapy Resistance by the Induction of Ferroptosis. Drug Resist. Updat..

[14] Lei G., Zhuang L., Gan B. (2022). Targeting Ferroptosis as a Vulnerability in Cancer. Nat. Rev. Cancer.

[15] Campbell N.K., Fitzgerald H.K., Dunne A. (2021). Regulation of Inflammation by the Antioxidant Haem Oxygenase 1. Nat. Rev. Immunol..

[16] Yu F., Wang Z., Zhang Z., Zhou J., Li J., Chen J., Du G., Zhao X. (2024). Biosynthesis, Acquisition, Regulation, and Upcycling of Heme: Recent Advances. Crit. Rev. Biotechnol..

[17] Chiabrando D., Vinchi F., Fiorito V., Mercurio S., Tolosano E. (2014). Heme in Pathophysiology: A Matter of Scavenging, Metabolism and Trafficking across Cell Membranes. Front. Pharmacol..

[18] Swenson S.A., Moore C.M., Marcero J.R., Medlock A.E., Reddi A.R., Khalimonchuk O. (2020). From Synthesis to Utilization: The Ins and Outs of Mitochondrial Heme. Cells.

[19] Kim H.J., Khalimonchuk O., Smith P.M., Winge D.R. (2012). Structure, Function, and Assembly of Heme Centers in Mitochondrial Respiratory Complexes. Biochim. Biophys. Acta..

[20] Kelland L. (2007). The Resurgence of Platinum-Based Cancer Chemotherapy. Nat. Rev. Cancer.

[21] Jordan M.A., Wilson L. (2004). Microtubules as a Target for Anticancer Drugs. Nat. Rev. Cancer.

[22] Pommier Y. (2006). Topoisomerase I Inhibitors: Camptothecins and Beyond. Nat. Rev. Cancer.

[23] Majeed U., Manochakian R., Zhao Y., Lou Y. (2021). Targeted Therapy in Advanced Non-Small Cell Lung Cancer: Current Advances and Future Trends. J. Hematol. Oncol..

[24] Forde P.M., Chaft J.E., Smith K.N., Anagnostou V., Cottrell T.R., Hellmann M.D., Zahurak M., Yang S.C., Jones D.R., Broderick S. (2018). Neoadjuvant PD-1 Blockade in Resectable Lung Cancer. N. Engl. J. Med..

[25] Guo J., Xu B., Han Q., Zhou H., Xia Y., Gong C., Dai X., Li Z., Wu G. (2018). Ferroptosis: A Novel Anti-Tumor Action for Cisplatin. Cancer Res. Treat..

[26] Liu F., Tang L., Liu H., Chen Y., Xiao T., Gu W., Yang H., Wang H., Chen P. (2024). Cancer-Associated Fibroblasts Secrete FGF5 to Inhibit Ferroptosis to Decrease Cisplatin Sensitivity in Nasopharyngeal Carcinoma through Binding to FGFR2. Cell Death Dis..

[27] Chen Y., Zhou H., Yang S., Su D. (2021). Increased ABCC2 Expression Predicts Cisplatin Resistance in Non-Small Cell Lung Cancer. Cell Biochem. Funct..

[28] Hooda J., Shah A., Zhang L. (2014). Heme, an Essential Nutrient from Dietary Proteins, Critically Impacts Diverse Physiological and Pathological Processes. Nutrients.

[29] Bonkovsky H.L., Guo J.-T., Hou W., Li T., Narang T., Thapar M. (2013). Porphyrin and Heme Metabolism and the Porphyrias. Compr. Physiol..

[30] Wang J.-Q., Yang Y., Cai C.-Y., Teng Q.-X., Cui Q., Lin J., Assaraf Y.G., Chen Z.-S. (2021). Multidrug Resistance Proteins (MRPs): Structure, Function and the Overcoming of Cancer Multidrug Resistance. Drug Resist. Updates.

[31] Mao Y.-X., Chen Z.-P., Wang L., Wang J., Zhou C.-Z., Hou W.-T., Chen Y. (2024). Transport Mechanism of Human Bilirubin Transporter ABCC2 Tuned by the Inter-Module Regulatory Domain. Nat. Commun..

[32] Zhou N., Yuan X., Du Q., Zhang Z., Shi X., Bao J., Ning Y., Peng L. (2023). FerrDb V2: Update of the Manually Curated Database of Ferroptosis Regulators and Ferroptosis-Disease Associations. Nucleic Acids Res..

[33] Wang Y., Gan X., Cheng X., Jia Y., Wang G., Tang X., Du H., Li X., Liu X., Xing X. (2024). ABCC2 Induces Metabolic Vulnerability and Cellular Ferroptosis via Enhanced Glutathione Efflux in Gastric Cancer. Clin. Transl. Med..

[34] Meyer M.-L., Fitzgerald B.G., Paz-Ares L., Cappuzzo F., Jänne P.A., Peters S., Hirsch F.R. (2024). New Promises and Challenges in the Treatment of Advanced Non-Small-Cell Lung Cancer. Lancet.

[35] Zou Y., Xie J., Zheng S., Liu W., Tang Y., Tian W., Deng X., Wu L., Zhang Y., Wong C.-W. (2022). Leveraging Diverse Cell-Death Patterns to Predict the Prognosis and Drug Sensitivity of Triple-Negative Breast Cancer Patients after Surgery. Int. J. Surg..

